# Association analysis of dopaminergic degeneration and the neutrophil-to-lymphocyte ratio in Parkinson’s disease

**DOI:** 10.3389/fnagi.2024.1377994

**Published:** 2024-04-08

**Authors:** Fengjiao Zhang, Bin Chen, Wenhua Ren, Yayun Yan, Xiaoqi Zheng, Shuxian Jin, Ying Chang

**Affiliations:** ^1^Departments of Neurology, China-Japan Union Hospital, Jilin University, Changchun, China; ^2^Departments of Nuclear Medicine, China-Japan Union Hospital, Jilin University, Changchun, China

**Keywords:** Parkinson’s disease, dopaminergic degeneration, neutrophil-to-lymphocyte, peripheral inflammation, peripheral inflammatory responses

## Abstract

**Introduction:**

Peripheral inflammatory responses are suggested to play a major role in the pathophysiology of Parkinson’s disease (PD). The neutrophil-to-lymphocyte ratio (NLR), a new recognized biomarker, can reflect peripheral inflammation in PD. However, the association between the NLR and dopaminergic degeneration in PD remains unclear.

**Methods:**

In this retrospective study, 101 enrolled PD patients were categorized into early-stage and advanced-stage PD based on the Hoehn and Yahr (HY) scale. We evaluated the clinical characteristics, peripheral immune profile, and 11C-CFT striatal dopamine transporter (DAT) binding levels. Linear regression analyses were employed to assess the associations between NLR and striatal DAT levels at different stages in PD patients.

**Results:**

Covariate-controlled regression analysis revealed that higher NLR was significantly associated with lower DAT levels in the caudate (β = −0.27, *p* = 0.003) and the putamen (β = −0.27, *p* = 0.011). Moreover, in the early-stage PD subgroup, a similar association was observed (caudate: β = −0.37, *p* = 0.013; putamen: β = −0.45, *p* = 0.005). The lymphocytes count was correlated positively with the striatal DAT levels in the Spearman correlation analysis whether in total patients (caudate: ρ = 0.25, *p* = 0.013; putamen: ρ = 0.22, *p* = 0.026) or in the early-stage subgroup (caudate: ρ = 0.31, *p* = 0.023, putamen: ρ = 0.34, *p* = 0.011).

**Conclusion:**

Dopaminergic degeneration is associated with peripheral inflammation in PD. The NLR, a widely used inflammatory marker, may have the potential to reflect the degree of dopaminergic degeneration in individuals with early-stage PD.

## 1 Introduction

Parkinson’s disease (PD) is a common progressive neurodegenerative disorder characterized by impairment of the dopaminergic neurons in the substantia nigra pars compacta (SNpc). With the neurodegeneration of the SNpc, dopamine depletion is observed through the nigrostriatal pathway, which is primarily responsible for the motor symptoms of PD. Dopamine transporter (DAT) is a membrane protein located on the presynaptic membrane of central dopaminergic neurons. Its primary function is to reuptake excess dopamine in the synaptic cleft. It is a crucial regulatory factor that reflects the extent of dopaminergic degeneration in PD (Cheng and Bahar, 2019). DAT can be evaluated using 11C-CFT positron emission tomography/computed tomography (PET/CT), a molecular imaging technique that enables the utilization of validated automated or semi-automated neuroimaging methods to measure density in specific regions of interest, including the caudate and putamen (Labrador-Espinosa et al., 2021; Nozaki et al., 2021).

Regarding the neuropathological characteristics of PD, the existence of aggregated misfolded α-synuclein inclusions, commonly referred to as Lewy bodies, has been observed in the SNpc along with signs of inflammation (Moore et al., 2005). Recent evidence suggests that both neuroinflammation and peripheral inflammation play significant roles in the onset and progression of PD. Concerning neuroinflammation, pioneering research by McGeer initially established the connection between neuroinflammation and PD. He observed activated microglial infiltrations in postmortem brains obtained from PD patients (McGeer et al., 1988). In a meta-analysis of human transcriptomics, Noori et al. also revealed that neuroinflammation is a shared characteristic among various neurodegenerative diseases such as Lewy body diseases (LBD) and other atypical parkinsonisms (Noori et al., 2021). Additionally, evidence from both animal models and post-mortem studies of PD has indicated abnormal over-expression of activated microglia and astrocytes in the affected regions of the brain, primarily in the SNpc (Gu et al., 2010; Colonna and Butovsky, 2017). Moreover, there is increasingly compelling evidence suggesting that peripheral inflammation is involved in the early pathophysiology of PD and undergoes dynamic changes throughout the course of the disease (Pajares, 2020).

The neutrophil-to-lymphocyte ratio (NLR), a simple ratio derived from the counts of neutrophils and lymphocytes in peripheral blood, offers a significant advantage by encompassing the two key immune response pathways. Neutrophils serve as indicators of the innate immune response, whereas lymphocytes signify the adaptive immune response and complex immune regulation processes (Song et al., 2021; Buonacera et al., 2022). The NLR, a widely utilized biomarker reflecting peripheral inflammation, has undergone preliminary validation concerning its association with neurodegenerative diseases. Previous studies have demonstrated a significant increase in the NLR in patients with multiple system atrophy (MSA) and Alzheimer’s disease (AD) (Kuyumcu et al., 2012; Zhang et al., 2022). Recent researches have also reported changes in the NLR observed in patients with PD (Sanjari Moghaddam et al., 2018; Muñoz-Delgado et al., 2021). However, it is still unclear whether this peripheral biomarker can accurately reflect the progression of pathological changes, primarily related to DAT density in PD. Moreover, it is also unknown whether NLR could serve as an early indicator of the severity of dopaminergic degeneration in PD patients. Therefore, we performed the current study to elucidate the association between peripheral inflammation and striatal DAT levels in PD patients at different clinical stages.

## 2 Methods

### 2.1 Subjects and clinical assessment

We retrospectively enrolled 101 PD patients admitted to the department of Neurology, China-Japan Union Hospital of Jilin University from March 2019 to July 2023. According to Hoehn & Yahr scale, patients were categorized into the early-stage (grades 1 ~ 2.5, *n* = 55) and advanced-stage PD (grades 3 ~ 5, *n* = 46). Inclusion criteria were listed as follows: diagnosed as PD according to the 2015 version of Movement Disorder Society Clinical Diagnostic Criteria and patients were able to cooperate with the evaluation process using relevant scales (Postuma et al., 2015). Exclusion criteria were: (1) clinical diagnosis of Parkinsonism-Plus syndrome or Parkinsonian syndrome caused by cerebrovascular diseases, encephalitis and other etiologies; (2) chronic wasting diseases that can influence peripheral inflammation such as active infections or malignancies; (3) combined with autoimmune diseases or currently undergoing immunotherapy; (4) combined with other neurological diseases and mental diseases. This study was approved by the Ethics Committee of China-Japan Union Hospital of Jilin University. The Movement Disorder Society-Sponsored Revision of the Unified Parkinson’s Disease Rating Scale part III (MDS-UPDRS-III) and H&Y scale were used to assess the severity of the disease. Montreal Cognitive Assessment (MOCA) was used to evaluate global cognition function. Levodopa equivalent daily dose (LEDD) was calculated based on the records of dopaminergic treatment and previous guidelines (Tomlinson et al., 2010).

### 2.2 Neuroimaging processing and DAT quantification

The imaging acquisition of subjects was performed following standardized imaging protocols of 11C-CFT PET/CT. All subjects were instructed to withhold anti-Parkinson’s medications for at least 12 h before scanning. The subjects were injected intravenously with 10 mCi (370 Mbq) of 11C-CFT, and 30 min following the injection, DAT images were acquired in three-dimensional mode over 20 min. Subsequently, 11C-CFT PET/CT images were spatially normalized using SPM12 (Wellcome Department of Imaging Neuroscience, Institute of Neurology, UCL, London, UK) running under MATLAB 2023a (The MathWorks, Natick, MA) (The MathWorks Inc. 2023) and then transformed into the standard Montreal Neurological Institute (MNI) space (Tzourio-Mazoyer et al., 2002; García-Gómez et al., 2013). After smoothing, obtained images were re-segmented and co-registered to the AAL3 template, with a voxel size of 4 × 4 × 4 mm (Rolls et al., 2020). Finally, the images were converted to standardized uptake value ratio (SUVR) units by normalizing each brain voxel in relation to the occipital lobe. DAT levels were quantified by calculating the mean SUVR in regions of interest within the striatum (caudate and putamen).

### 2.3 Peripheral immune cell measurements

Peripheral blood samples were collected from the subjects and measured for neutrophils and lymphocytes using the BC-6900 hematology analyzer in our center’s laboratory. It utilizes the combination of impedance-based flow cytometry, laser scattering, and fluorescence staining for cells determination and analysis. The NLR was calculated as the ratio between the absolute neutrophil count and the absolute lymphocyte count.

### 2.4 Statistical analysis

All statistical analyses were performed using SPSS 27.0 software. The normality of the distribution of the measurement data was assessed by the Shapiro–Wilk test and normally distributed variables were presented as mean ± standard deviation (mean ± SD). Non-normally distributed data were reported as medians with interquartile ranges (IQR). Associations between peripheral immune cells (lymphocytes, neutrophils, and the NLR) and DAT levels in striatal regions (caudate and putamen) were evaluated through correlation analyses using Spearman’s correlation. Significant associations between peripheral inflammation and striatal DAT were further assessed using multivariate linear regression analyses, while controlling for sex, age, disease duration, LEDD, MDS-UPDRS III, and MOCA. Results from covariate-controlled regression models were expressed as coefficient (β), 95% confidence interval (95% CI), and *p* value (P). In this analysis, *p* < 0.05 was considered significant statistically.

## 3 Results

### 3.1 Clinical and demographic characteristics

A total of 101 patients with PD were enrolled in this study and divided into early-stage (grades 1–2.5, *n* = 55) and advanced-stage PD (grades 3–5, *n* = 46). The clinical and demographic characteristics of patients were summarized in Table 1.

**Table 1:** Demographic and clinical characteristics of patients with PD.

Characteristics	Total cohort (*n* = 101)	Early-stage (*n* = 55)	Advanced-stage (*n* = 46)
Age (year, mean ± SD)	66.91 ± 8.18	65.05 ± 8.25	69.13 ± 7.59
Sex (male, %)	58 (57.4)	36 (65.5)	22 (47.8)
Age of onset (year, mean ± SD)	61.02 ± 8.07	60.40 ± 8.36	61.76 ± 7.74
Disease duration (year, IQR)	6.00 (3.00–8.00)	4.00 (2.00–7.00)	7.00 (5.00–10.00)
LEDD (mg, IQR)	300.00 (0–568.75)	300.00 (0–437.50)	456.25 (75.00–718.75)
HY in OFF state (IQR)	2.50 (2.00–3.00)	2.00 (1.50–2.00)	3.00 (3.00–4.00)
MDS-UPDRS-III score (off stage, mean ± SD)	49.89 ± 22.78	35.80 ± 18.24	65.68 ± 16.04
MOCA score (IQR)	21.00 (17.00–24.00)	22.00 (20.00–25.00)	17.00 (14.00–21.00)
Lymphocytes (×10^9^/L, mean ± SD)	1.56 ± 0.42	1.55 ± 0.45	1.57 ± 0.38
Neutrophils (×10^9^/L, IQR)	3.29 (2.57–4.13)	3.00 (2.58–3.98)	3.64 (2.56–4.81)
NLR (IQR)	2.06 (1.64–2.81)	1.95 (1.62–2.51)	2.19 (1.79–3.00)
SUVR score in caudate (mean ± SD)	1.43 ± 0.39	1.47 ± 0.40	1.38 ± 0.37
SUVR score in putamen (IQR)	1.79 (1.71–1.98)	1.83 (1.74–2.02)	1.75 (1.68–1.89)

LEDD, Levodopa equivalent daily dose; HY, Hoehn and Yahr stage; MDS-UPDRS-III, Movement Disorders Society Unified Parkinson’s Disease Rating Scale part III; MOCA, Montreal Cognitive Assessment; NLR, neutrophil-to-lymphocyte ratio; SUVR, standardized uptake value ratio.

### 3.2 Spearman’s correlations between the peripheral immune response and striatal dopamine transporter

In the total cohort with PD, DAT levels in the caudate and putamen exhibited a significant negative spearman correlation with the NLR (ρ = −0.29, *p* = 0.003 and ρ = −0.28, *p* = 0.004, respectively), as well as a positive correlation with the lymphocyte count (ρ = 0.25, *p* = 0.013 and ρ = 0.22, *p* = 0.026, respectively). However, no correlation was observed between striatal DAT levels (mainly in the caudate and putamen) and the neutrophil count (ρ = −0.17, *p* = 0.088 and ρ = −0.20, *p* = 0.051, respectively).

**Figure 1:**
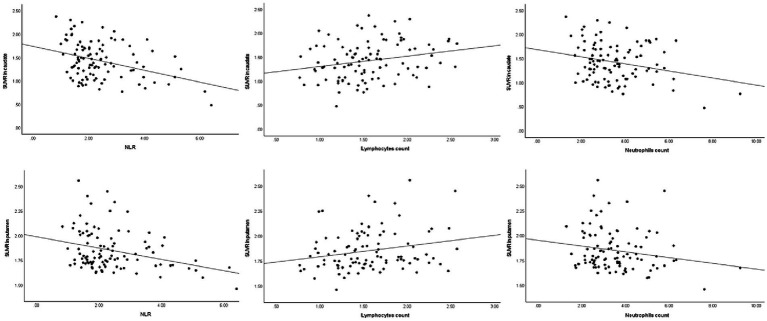
Spearman’s correlations between striatal DAT and the peripheral immune response in total PD patients. DAT, dopamine transporter; NLR, neutrophil-to-lymphocyte ratio; SUVR, standardized uptake value ratio.

**Figure 2:**
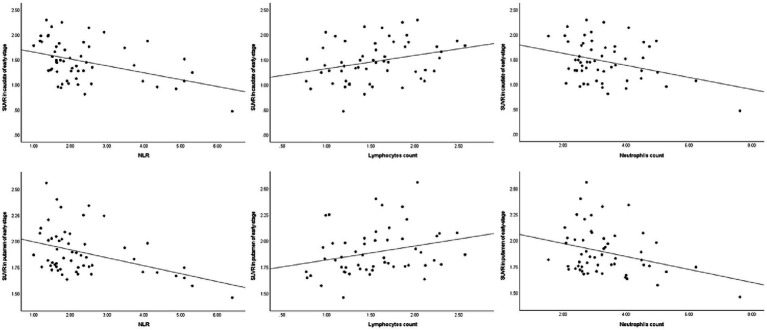
Spearman’s correlations between striatal DAT and the peripheral immune response in early-stage PD. DAT, dopamine transporter; NLR, neutrophil-to-lymphocyte ratio; SUVR, standardized uptake value ratio.

**Figure 3:**
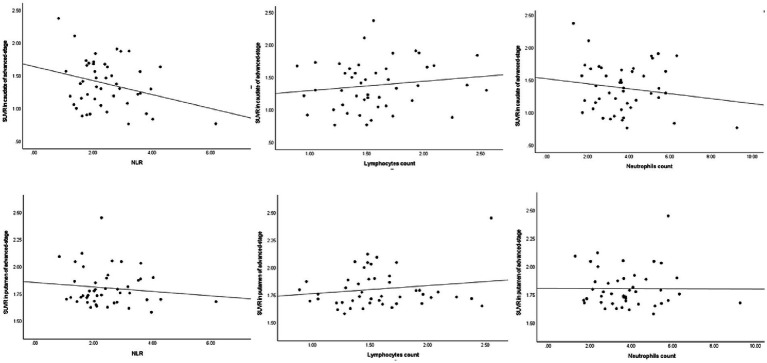
Spearman’s correlations between striatal DAT and the peripheral response in advanced-stage PD. DAT, dopamine transporter; NLR, neutrophil-to-lymphocyte ratio; SUVR, standardized uptake value ratio.

Similarly, in early-stage PD patients, the same correlations were observed, with a significant negative correlation between DAT levels in the caudate and putamen and the NLR (ρ = −0.41, *p* = 0.002 and ρ = −0.38, p = 0.004, respectively), as well as a significant positive correlation with lymphocyte count (ρ = 0.31, *p* = 0.023 and ρ = 0.34, *p* = 0.011, respectively). Once again, there was no significant correlation between striatal DAT levels (mainly in the caudate and putamen) and neutrophil count in early-stage PD patients (ρ = −0.21, *p* = 0.119 and ρ = −0.22, *p* = 0.107, respectively). As for advanced-stage PD patients, no correlations were found between DAT levels in the striatum (mainly in the caudate and putamen) and the NLR, lymphocyte count or neutrophil count. The correlation between the peripheral immune inflammation index of patients and the regions of interest regarding striatal DAT levels is shown in Figures 1–3 and Table 2.

**Table 2:** Spearman’s rank order analyses between SUVR in striatal regions-of-interest and the peripheral immune profile in patients with PD.

	Total cohort (*n* = 101)				Early-stage (*n* = 55)				Advanced-stage (*n* = 46)			
	SUVR in caudate		SUVR in putamen		SUVR in caudate		SUVR in putamen		SUVR in caudate		SUVR in putamen	
	ρ	P	ρ	P	ρ	P	ρ	P	ρ	P	ρ	P
NLR	−0.29	0.003	−0.28	0.004	−0.41	0.002	−0.38	0.004	−0.15	0.338	−0.06	0.700
Lymphocytes count	0.25	0.013	0.22	0.026	0.31	0.023	0.34	0.011	0.15	0.322	0.08	0.619
Neutrophils count	−0.17	0.088	−0.20	0.051	−0.21	0.119	−0.22	0.107	−0.07	0.662	−0.04	0.770

NLR neutrophil-to-lymphocyte ratio; SUVR standardized uptake value ratio. ρ Spearman’s correlation coefficient; P p-value.

### 3.3 Associations between the peripheral immune response and striatal dopamine transporter

In the univariate regression models of the total PD patients, lower DAT levels in the caudate and putamen were significantly associated with higher NLR (β = −0.37, *p* < 0.001 and β = −0.31, *p* = 0.001, respectively). As for early-stage PD patients, we similarly found that lower DAT levels in the caudate and putamen were associated with a higher NLR (β = −0.41, *p* = 0.002 and β = −0.41, p = 0.002, respectively) and a lower lymphocyte count (β = 0.30, *p* = 0.028 and β = 0.27, *p* = 0.048, respectively) in the univariate linear regression models.

These associations about the NLR were also supported in covariate-controlled regression models. Lower DAT levels in the caudate and putamen remained significantly associated with higher NLR (β = −0.27, *p* = 0.003 and β = −0.27, *p* = 0.011, respectively) in the total PD patients. Regarding the early-stage PD patients, it demonstrated that lower DAT levels in both the caudate and putamen were also associated with higher NLR (β = −0.37, *p* = 0.013 and β = −0.45, *p* = 0.005, respectively). But the association about lymphocyte count was not found in covariate-controlled regression models whether in early-stage or advanced-stage PD patients.

However, no association was found with the neutrophil count in regression models (whether in the univariate or covariate-controlled regression models). The results are presented in Table 3.

**Table 3:** Regression analyses between SUVR in striatal regions-of-interest and the peripheral immune profile in patients with PD.

	Total cohort (*n* = 101)						Early-stage (*n* = 55)					
	SUVR in caudate			SUVR in putamen			SUVR in caudate			SUVR in putamen		
	β	95%CI	P	β	95%CI	P	β	95%CI	P	β	95%CI	P
Unadjusted												
NLR	−0.37	(−0.19 ~ −0.06)	<0.001	−0.31	(−0.09 ~ −0.02)	0.001	−0.41	(−0.22 ~ −0.05)	0.002	−0.41	(−0.12 ~ −0.03)	0.002
Lymphocytes count	0.23	(0.04 ~ 0.39)	0.019	0.22	(0.01 ~ 0.20)	0.027	0.30	(0.03 ~ 0.50)	0.028	0.27	(0.01 ~ 0.26)	0.048
Neutrophils count	−0.25	(−0.13 ~ −0.02)	0.011	−0.19	(−0.06 ~ −0.01)	0.058	−0.33	(−0.22 ~ −0.03)	0.013	−0.31	(−0.12 ~ −0.01)	0.021
Adjusted												
NLR	−0.27	(−0.16 ~ −0.03)	0.003	−0.27	(−0.09 ~ −0.01)	0.011	−0.37	(−0.22 ~ −0.03)	0.013	−0.45	(−0.14 ~ −0.03)	0.005
Lymphocytes count	0.11	(−0.07 ~ 0.26)	0.250	0.16	(−0.02 ~ 0.18)	0.123	0.20	(−0.08 ~ 0.41)	0.173	0.28	(−0.01 ~ 0.28)	0.069
Neutrophils count	−0.19	(−0.11 ~ −0.01)	0.037	−0.15	(−0.06 ~ −0.01)	0.144	−0.24	(−0.19 ~ 0.01)	0.081	−0.22	(−0.11 ~ 0.02)	0.132

SUVR, standardized uptake value ratio; NLR, neutrophil-to-lymphocyte ratio. β estimate of coefficient; CI confidence interval; P p-value. Unadjusted: data are obtained by univariate regression analyses between SUVR in striatal regions-of-interest and the peripheral immune profile. Adjusted: data are obtained by covariate-controlled regression analyses with SUVR as the dependent variable, and adjusted for age, sex, disease duration, levodopa equivalent daily dose and Montreal Cognitive Assessment.

## 4 Discussion

In this study, our main findings are as follows: (1) striatal dopaminergic degeneration is significantly associated with NLR; (2) in early-stage PD patients, the level of striatal DAT is significantly negatively correlated with NLR, and as PD progresses, NLR significantly increases. However, in advanced-stage PD patients, NLR has no significant predictive value for changes in DAT levels; (3) in the Spearman correlation analysis, the lymphocytes count was correlated positively with the striatal DAT levels; (4) association between the striatal DAT levels and the neutrophil count was not clearly observed.

To date, the majority of researchers support the involvement of peripheral inflammation in the development of PD. Brodacki et al. reported abnormal elevations in IL2, IL6, and TNF-α levels in PD patients, suggesting a potential link between peripheral inflammation and PD (Brodacki et al., 2008). Furthermore, peripheral inflammation may be also associated with other atypical parkinsonisms and neurodegenerative diseases, including Alzheimer’s disease (AD) and PSP (Akiyama et al., 2000; Madetko-Alster et al., 2023). However, the specific molecular mechanisms remain largely unclear. NLR, as a non-invasive and convenient indicator of peripheral inflammation, has been preliminarily established to be associated with PD in previous studies. In a case–control study, researchers demonstrated that patients with PD had a significantly higher NLR in peripheral blood compared to healthy controls (Muñoz-Delgado et al., 2021). Moreover, in a large sample of PD patients with different motor subtypes, NLR exhibited a negative correlation to striatal binding ratio (SBR) in the bilateral putamen and ipsilateral caudate among tremor dominant PD individuals (Sanjari Moghaddam et al., 2018). Compared to neuroinflammation, researchers may place greater emphasis on peripheral inflammation because there is a growing interest in the possibility that PD may have its initial origins in peripheral inflammation (Pajares, 2020). In addition, researchers emphasized that peripheral inflammation can affect the brain through the blood–brain barrier (BBB) or the autonomic nervous system, further promoting microglia into an “active” state and triggering an ongoing neurodegenerative changes (Pajares, 2020). In our study, we founded a negative correlation between the level of striatal DAT and NLR in the overall group of PD patients. When we divided the total patients into early-stage and advanced-stage subgroups, we observed a similar finding in the early-stage PD subgroup. The level of striatal DAT is significantly and negatively correlated with NLR in early-stage PD patients. This finding is also consistent with a recent literature report. Researchers enrolled 344 *de novo* patients with PD from an international, prospective, longitudinal cohort study named the Parkinson’s Progression Markers Initiative (PPMI) to explore the association about peripheral inflammation and dopaminergic degeneration. The results demonstrated a negative correlation between striatal dopaminergic degeneration and NLR in newly diagnosed PD patients (Muñoz-Delgado et al., 2023).

Nevertheless, in our study, NLR showed no significant association with DAT levels in advanced-stage PD patients. This outcome implies that NLR, as a widely employed peripheral inflammatory marker, might possess a greater predictive value for the degenerative changes seen in PD, particularly in the earlier stages of the disease. However, there is limited research that specifically combines peripheral inflammatory markers with PET/CT to evaluate the relationship between the inflammatory response and *in vivo* neurodegeneration. Additionally, there is a scarcity of studies investigating peripheral inflammatory markers in PD patients based on different stages of the disease. To the best of our knowledge, this was the first study to analyze separately the contributions of peripheral inflammatory cells to striatal DAT levels dividing into different stages. Our results indicated a negative correlation between the level of striatal DAT and the NLR in early-stage PD patients, and as PD progresses, NLR significantly increases. This suggests that the NLR may have the potential to indicate the extent of dopaminergic degeneration in individuals with early-stage PD.

Clinical research have consistently found a decrease in lymphocyte count in PD patients, which is also the reason for the increase in NLR (Tansey and Romero-Ramos, 2019; Pajares, 2020; Grillo et al., 2023). Moreover, a recent study reported that a lower lymphocyte count was associated with an increased risk of subsequent PD (Jensen et al., 2021). This may be attributed to the chronic inflammation-induced disruption and increased permeability of the BBB. Subsequently, the migration of a significant number of lymphocytes into the central nervous system (CNS) further exacerbates the breakdown of the BBB, allowing more lymphocytes to infiltrate the CNS (Pajares, 2020). This phenomenon suggests that inflammatory responses may play a role in forming a vicious cycle during the progression of PD. Although we did not observe similar changes in lymphocyte counts in our multiple linear regression model, the correlation was evident in Spearman correlation analysis, particularly in early-stage patients with PD. This may be attributed to the limited sample size in our study, which may have hindered the demonstration of this correlation in relation to disease staging. Further longitudinal and targeted research is needed to comprehensively evaluate the role of lymphocytes about PD in the future. In contrast to lymphocyte count, we did not find an association between neutrophil counts and striatal DAT levels in our study. This could be demonstrated to a notable increase in neutrophils primarily predominantly occurring during the initial years before the diagnosis of PD (Terkelsen et al., 2022). As the disease progresses, this subtle effect may only contribute to maintaining a chronic inflammatory state in PD (Klüter et al., 1995; Terkelsen et al., 2022). Currently, there is limited research focused on investigating the role of neutrophils in the progression of PD. Hence, further studies are required to provide a deeper understanding of the impact of neutrophils on the pathogenesis of PD.

The main limitation of our study is its retrospective design, which may inherently introduce potential confounding variables that may affect the results. However, we addressed this issue by implementing rigorous exclusion criteria and performing multivariate adjusted statistical analysis to minimize the influence of confounding factors. Besides, some chronic inflammatory diseases may influence the study. Those diseases in our study have been excluded mainly based on the medical history provided by patients and their families. However, the absence of relevant laboratory indicators may exert an influence on the experimental outcomes. In our subsequent studies, we intend to introduce hematological indicators related to inflammation, aiming to exclude potential subjects that may potentially affect the results. Furthermore, our study had a small sample size, which prevented us from conducting a comprehensive study of the different motor subtypes of PD. However, this gap provides a key area for future research. Even so, NLR may still has the potential to be a non-invasive biomarker for early diagnosis of PD. Although further studies are needed to explore the pathophysiological role of NLR and its potential in the differential diagnosis of other neurodegenerative diseases, the combination of NLR with clinical and molecular imaging techniques can enhance the diagnostic decision in PD.

## 5 Conclusion

Dopaminergic degeneration is associated with peripheral inflammation in PD. The NLR, a widely used inflammatory marker, may have the potential to reflect the degree of dopaminergic degeneration in individuals with early-stage PD.

## Data availability statement

The raw data supporting the conclusions of this article will be made available by the authors, without undue reservation.

## Ethics statement

The studies involving humans were approved by Ethics Committee of the China-Japan Union Hospital of Jilin University. The studies were conducted in accordance with the local legislation and institutional requirements. The ethics committee/institutional review board waived the requirement of written informed consent for participation from the participants or the participants’ legal guardians/next of kin because the waiver of informed consent is justified in this research as it involves the use of identifiable personal biological samples or data, and it is not feasible to locate the participants for re-consent. Additionally, the study has implemented adequate measures to ensure the protection of personal information and does not involve any invasion of personal privacy or commercial interests.

## Author contributions

FZ: Conceptualization, Data curation, Investigation, Methodology, Visualization, Writing – original draft. BC: Conceptualization, Data curation, Investigation, Methodology, Visualization, Writing – original draft. WR: Software, Validation, Writing – review & editing. YY: Conceptualization, Methodology, Supervision, Writing – review & editing. XZ: Software, Validation, Writing – review & editing. SJ: Software, Validation, Writing – review & editing. YC: Conceptualization, Methodology, Supervision, Writing – review & editing.
